# Evidence for systematic autopsies in COVID-19 positive deceased: Case report of the first German investigated COVID-19 death

**DOI:** 10.1007/s00194-020-00401-4

**Published:** 2020-05-25

**Authors:** A. Fitzek, J. Sperhake, C. Edler, A. S. Schröder, A. Heinemann, F. Heinrich, A. Ron, H. Mushumba, M. Lütgehetmann, K. Püschel

**Affiliations:** 1 Institute of Legal Medicine, University Medical Center Hamburg-Eppendorf, Hamburg, Germany; 2 Institute for Medical Microbiology, Virology and Hygiene, University Medical Center Hamburg-Eppendorf, Hamburg, Germany

**Keywords:** SARS-CoV‑2, COVID-19, High risk autopsy, Deceased, Biological Agents Ordinance, SARS-CoV‑2, COVID-19, Hochrisiko-Autopsie, Verstorbene, Biostoffverordnung

## Abstract

Forensic medicine and pathology involve specific health risks, whereby health workers are dealing with microorganisms, cells or parasites, which are referred to as biological agents. Biological agents are divided into four categories according to § 3 of the Biological Agents Ordinance. The newly identified coronavirus, severe acute respiratory syndrome, coronavirus 2 (SARS-CoV-2) that has spread rapidly around the world is placed into category 3 of the Biological Agents Ordinance, meaning pathogens that can cause serious illnesses in humans and may pose a risk to workers. The Robert Koch Institute, the German government’s central scientific institution in the field of biomedicine issued the announcement, that aerosol-producing measures (including autopsies) of SARS-CoV‑2 infected bodies should be avoided, despite the fact that autopsies are an important source of understanding the pathomorphological course of new diseases. The first German case of death due to a proven SARS-CoV‑2 infection is presented with global multifocal reticular consolidation in the post-mortem computed tomography (CT) scan, a macroscopic and microscopic viral pneumonia and viral RNA of SARS-CoV‑2 in pharyngeal mucosa and lung tissue.

## Abstract (de)

Die Rechtsmedizin und die Pathologie bergen spezifische Gesundheitsrisiken, bei denen die Mitarbeiter mit Mikroorganismen arbeiten, die als biologische Arbeitsstoffe bezeichnet werden. Biologische Arbeitsstoffe werden gemäß § 3 der Biostoffverordnung in vier Kategorien eingeteilt. Das neu identifizierte, sich weltweit rasch ausgebreitende Coronavirus SARS-CoV‑2 (schweres akutes respiratorisches Syndrom, Corona-Virus 2) wird in Kategorie 3 der Verordnung über biologische Arbeitsstoffe eingestuft, d. h. Krankheitserreger, die beim Menschen schwere Krankheiten hervorrufen und eine Gefahr für die Arbeitnehmer darstellen können. Das Robert-Koch-Institut, die zentrale wissenschaftliche Einrichtung der Deutschen Regierung auf dem Gebiet der Biomedizin, gab die Ankündigung heraus, dass aerosolproduzierende Maßnahmen (einschließlich Sektionen) von SARS-CoV-2-infizierten Körpern vermieden werden sollten, wobei die Obduktion eines Verstorbenen eine wesentliche Quelle zum Verständnis eines pathomorphologischen Verlaufs von Krankheiten darstellt. Der erste deutsche SARS-CoV‑2 Todesfall wird vorgestellt. In der postmortalen Computertomographie (CT) zeigten sich eine globale Milchglastrübung beider Lungenflügel, autoptisch und histologisch eine virale Pneumonie und SARS-CoV‑2 virale RNA-Kopien in Rachenschleimhaut und Lungengewebe.

## Introduction

A persistent outbreak of pneumonia associated with severe acute respiratory syndrome coronavirus 2 (SARS-CoV-2) began in Wuhan, China in December 2019, a newly identified disease that has spread rapidly throughout Wuhan (Hubei Province) to other provinces in China and around the world [1]. The clinical spectrum of SARS-CoV‑2 pneumonia ranges from mild to severe cases. At this time there are no specific drugs available to treat SARS CoV‑2 infections [2].

To understand the pathomorphological course of SARS-CoV‑2, autopsies on infected deceased have to be performed. In this article the first German fatality due to a proven SARS-CoV-2-infection is presented.

## Case report

On Saturday 22 February 2020, the 59-year-old firefighter and his wife set off on the journey to Egypt. After a long delay at the airport in Hamburg they finally reached the hotel in Hurghada at 5:30 a.m. the next morning. Shortly after arrival, the journey continued with a 5‑h bus ride to Luxor, where they checked in on the Nile steamer at around 1:10 p.m. The long-awaited cruise vacation began the next morning with a visit to the Karnak and Luxor Temple and the Papyrus Gallery. They continued their journey over the Valley of the Kings, via Aswan to Abu Simbel. In the morning of the 28 February 2020 around 5:30 a.m. the man felt a sudden flickering of his eyes during the bus ride. Chills and dizziness accompanied his symptoms. He did not feel well the following days either. On Monday 2 March 2020, the man’s condition worsened and the bad general symptoms were accompanied by nocturnal chills, dizziness and coughing. Gastrointestinal symptoms were added as the day progressed. On the following day the couple contacted a doctor who prescribed antibiotics, cough syrup and paracetamol. The apparent improvement was followed a short time later by a marked deterioration in the man’s condition. Dizziness, coughing and general exhaustion put a strain on the holiday mood. The doctor was called in again and administered isotonic saline solution without improvement. The night of Friday was followed by admission to a local private clinic. High fever was added and a medical clarification and an X‑ray of the thorax were performed. Afterwards the wife was sent back to the hotel. The same night the man was transferred to a local hospital without contacting the wife. A smear test and a new X‑ray followed. The last time the wife saw her husband was on the evening of Friday 6 March 2020. Concern about the return to Germany, the critical hygiene conditions and the lack of feedback from medical staff in the hospital left the couple in the dark about the imminent drastic deterioration of the man’s condition. When the wife heard about the death of a German man in Hurghada in the news on Sunday 8 March 2020, she was already back in her home town in Germany. The news of her husband’s death was passed on to her that same evening by two police officers from the local police station. The man mentioned in the news was her husband. She first found out at this time that her husband had been transferred to another clinic in Hurghada as part of the further deterioration. For the following weeks she was put in enforced quarantine. The body of her husband was transferred to Germany with some delay. After the transfer, the embalmed body was taken to the Institute of Legal Medicine in Hamburg and examined there on the request of the relatives [3].

## Post-mortem CT scan

**Fig. 1:**
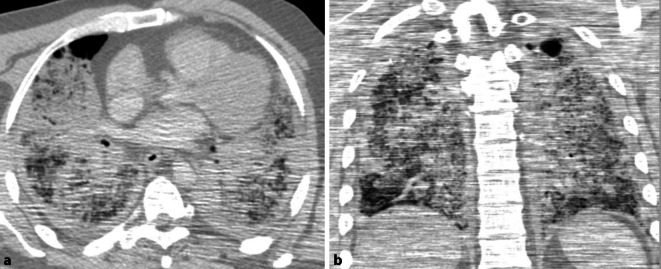
CT scans of the lungs in axial (**a**) and coronary (**b**) projections with bilateral pleural effusions, global multifocal reticular consolidation and subpleural milky glass opacities

The thoracic CT scan (axial/coronary projection) shows bilateral moderate pleural effusions and global multifocal reticular consolidation, especially in central areas (Fig. 1). Only circumscribed apical and ventral regions were excluded. Especially subpleural milky glass opacities with ground-glass density nodules could be detected; however, post-mortem artefacts, e.g. due to embalming, cannot be excluded in the context of pleural effusions and hypostasis.

## External post-mortem examination

The external post-mortem examination revealed a strongly built, slightly obese man, in good health and general condition. Signs of basic medical care, such as injection marks were found.

## Autopsy findings

**Fig. 2:**
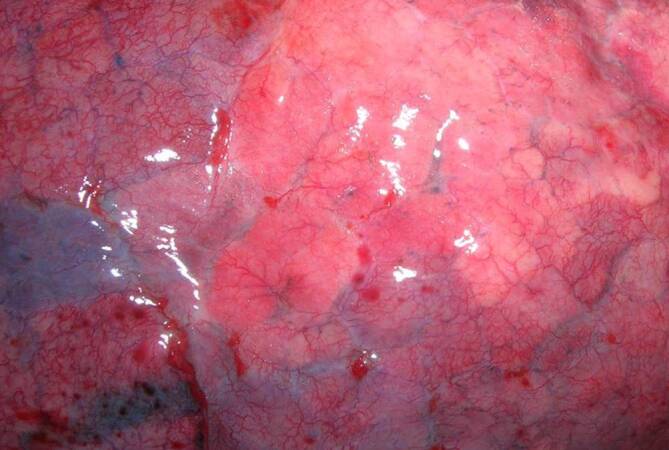
Macroscopic view of the surface of the lung showing grey-whitish pallor and fading of the vessels

**Fig. 3:**
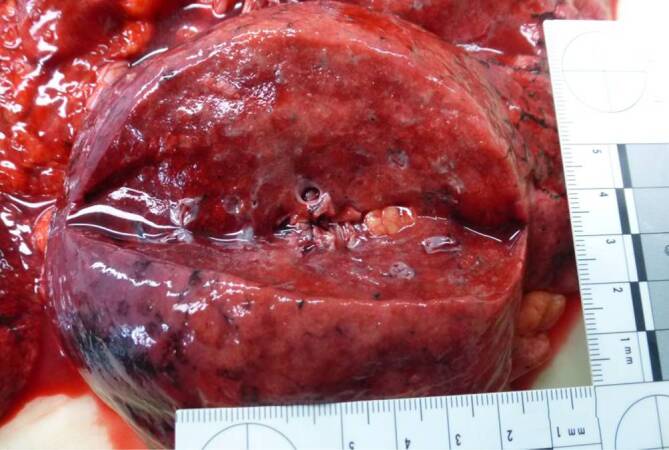
Macroscopic view of the cut sections of the lungs showing a pneumonia with greyish-yellow multifocal areas

**Fig. 4:**
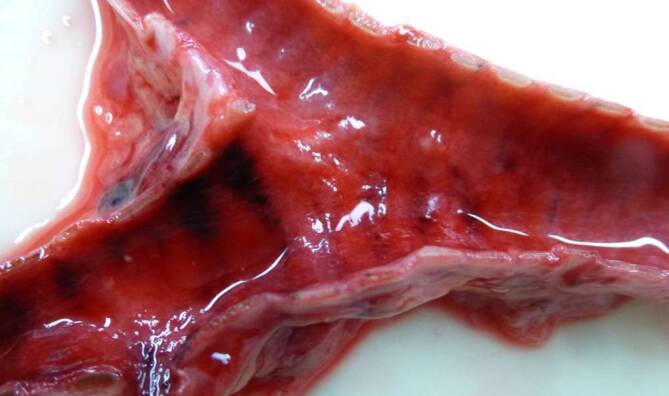
Macroscopic view of the tracheal bifurcation showing a hemorrhagic tracheobronchitis

The internal examination of the body revealed pneumonia superimposed by the embalming that had taken place in Egypt. The lungs were almost void of air, dark red in color and of increased consistency with greyish-yellow multifocal areas (Figs. 2 and 3) with a total lung weight of 1800 g. A distinct pulmonary edema with foamy secretion in the upper respiratory tract was found. The findings were accompanied by a hemorrhagic tracheobronchitis (Fig. 4). Additional diagnoses included a congestive cardiomyopathy with a heart weight of 600 g and a cor adiposum.

The cause of death was suspected to be viral pneumonia. The manner of death was defined as natural.

## Histological findings

**Fig. 5:**
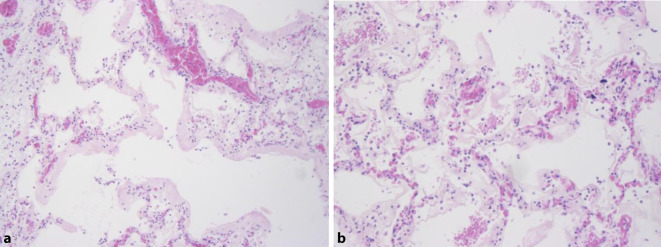
Hematoxylin and eosin staining of the lungs (**a**, **b**) with ubiquitous hyaline membranes, no granulocytic infiltrate (× 75)

Histology of the lung revealed ubiquitous hyaline membranes, vascular compressions and microthrombi in the sense of diffuse alveolar damage (Fig. 5). Beyond that additional protein-rich edema with low-grade lymphocyte infiltration were discovered. In the intestinal wall a moderate penetration by inflammatory cells was found. All other organs revealed no pathological findings.

## Virology findings

Regarding virology findings, a highly positive oropharygeal smear on SARS-CoV-2 was assessed. Similarly, the processing of organ tissue showed viral RNA in the pharyngeal mucosa (1.2 × 10^6^ viral copies of SARS-CoV-2) and the lungs (6.34 × 10^6^ viral copies of SARS-CoV-2). All other tissues tested showed negative results.

## Discussion

According to the Robert Koch Institute, there have been 5640 COVID-19 related deaths in Germany and 140 deaths in Hamburg (current state 26 April 2020).

In Hamburg all registered so-called corona deaths have been documented and autopsies were carried out at the Institute of Legal Medicine of the University Medical Center Hamburg-Eppendorf.

Judicial autopsies according to § 87 StPO (German Code of Criminal Procedure), forensic autopsies according to the Hamburg Autopsy Act, and autopsies ordered by the public health department according to § 25 (4) of the Infection Protection Law (IfSchG) are performed.

**Fig. 6:**
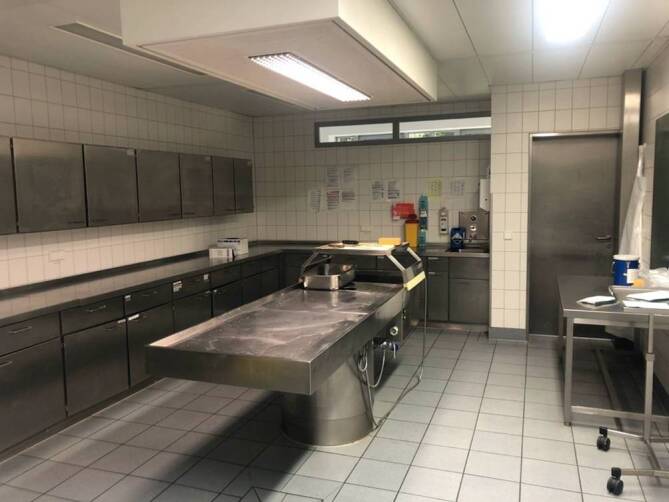
Autopsy room set-up. Extra ventilation, also underneath the autopsy table. Shelfes are empty

The autopsies are performed by two medical doctors and one technical assistant in an extra ventilated autopsy room. Protection for aerosol-producing measures on COVID-19 positive deceased are conducted: at least FFP2 face mask, eye and face protection (safety glasses/visor with protection at the top and sides), body protection with clean, long-sleeved, liquid-resistant or impermeable protective clothing and two gloves which allow sufficient overlap with the protective clothing [4]. The protective measures are left in the autopsy room and the shelves are emptied to be easily cleaned after the autopsy with antifect N liquid® (Schülke & Mayr GmbH, Norderstedt, Germany) (Fig. 6).

The Robert Koch Institute (RKI), the government’s central scientific institution in the field of biomedicine, issued the following public announcement regarding SARS-CoV‑2 positive corpses on 24 March 2020 [4]:

Internal post-mortem examinations, autopsies or other aerosol-producing measures should be avoided. If these are necessary, they should be kept to a minimum [4]*.*

At the beginning of April 2020 the Federal Association of German Pathologists (BDP), the German Society of Pathology (DGP) and the Federal Association of German Forensic Pathologists (BRDM) as well as the German Society of Forensic Pathology (DGRM) demanded as many autopsies of COVID-19 deceased as possible. In doing so, they also contradicted the recommendation of the Robert Koch Institute to avoid internal post-mortem examinations in these cases [5, 6]. This was opposed by other associations, such as the Royal College of Pathologists and other authors, which were critical about the autopsies of the deceased infected by SARS-CoV‑2 [7–9].

In the meantime, the RKI has reacted to the criticism and changed the controversial wording.

Special instructions for handling SARS-CoV‑2 infected deceased are given through the classification by the Committee on Biological Agents (ABAS). Biological agents are divided into four different risk groups according to their risk of infection. According to § 2 of the Biological Agents Ordinance, biological agents are microorganisms, including genetically modified microorganisms, cell cultures and human pathogenic endoparasites, which can cause infections or toxic effects in humans [10]. Biological agents also include transmissible agents associated with spongiform encephalopathy, which cause infections or transmissible diseases in humans [10].

Based on the available epidemiological data, the Committee for Biological Agents (ABAS) provisionally classified SARS-CoV‑2 into risk group 3 according to the Biological Agents Ordinance from a preventive point of view in its decision of 19 February 2020, and amended on 3 March 2020. Independent of national legal regulations, the risk of SARS-CoV‑2 infection must therefore be pointed out on the death certificate and it is recommended that COVID-19 is mentioned by name on the death or funeral certificate [11].

According to § 3 of the Biological Agents Ordinance (BioStoffV), biological agents are classified into four risk groups according to their risk of infection (Table 1; [10]).

**Table 1:** Risk categories of biological agents according to the Biological Agents Ordinance [10]

Risk category 1	Unlikely to cause diseases in humans
Risk category 2	Pathogens which can cause diseases in humans and could pose a risk to workers. It is unlikely to be spread in the population. Effective prevention or treatment is usually possible
Risk category 3	Pathogens which can cause serious diseases in humans and may pose a risk to workers. There may be a risk of spreading in the population. Normally an effective prevention or treatment is possible
Risk category 4	Pathogens which can cause serious illness in humans and pose a serious risk to workers. The risk of spreading in the population may be high. Normally, effective prevention or treatment is not possible

The BioStoffV applies to employees who may come into contact with infectious agents in the course of their work. The occupational health and safety regulations are specified in the Technical Rules for Biological Agents (TRBA), which are specific to the sector and topic. According to current knowledge, SARS-CoV‑2 can be transmitted by the inhalation of aerosols and by contact with mucous membranes (nose, mouth, eyes). On the basis of this knowledge, the necessary protective measures for activities to be carried out can be derived from the TRBA [12, 13]. The TRBA 250 and TRBA 100 regulate the measures for the protection of employees against infections in the healthcare and welfare sector and in laboratories [12, 13].

The decisive factor in determining the required level of protection is the assignment to targeted or non-targeted activities (see also § 5 BioStoffV) [10].

Targeted activities are directly aimed at a specific biological agent known to the species/subspecies level and the exposure of the employee is sufficiently known or can be estimated in the intended operation. A targeted activity is, for example, the reproduction of bacteria in pure culture or the reproduction of a defined virus species with the aid of cell cultures [12].

Non-targeted activities exist if one of the above criteria for targeted activities is not fulfilled. For example, the examination of human sample material (e.g. blood, swabs, tissue samples) within the framework of microbiological, clinicochemical or other special diagnostics is a non-targeted activity. This is also the case for activities involving sample material from a donor with clear suspicion of infection or positive results of infection, provided that these are not directed at the corresponding biological agent. Cytological or histological examinations of non-inactivated material are also non-targeted activities [12]. According to ABAS and TRBA 250, non-targeted activities, including autopsies can be carried out under the conditions of protection level 2 [11, 13].

The value of an autopsy, the associated pathomorphological and further virologic findings are crucial for the research of new diseases, the clinical course and treatment approaches for clinicians. In addition autopsies are also crucial to prevent further deaths and outbreaks, which is shown by a case report of a director of a mission hospital in Mango, Togo, with a suspected diagnosis of malaria tropica, typhoid fever and septic shock in an advanced stage, airlifted to Germany and admitted to the University Hospital in Cologne [14]. The diagnosis of a hemorrhagic fever virus, Lassa virus (category 4 of the BioStoffV) was made only through the requested autopsy and further histology and virologic findings [14]. The autopsy and further findings were life saving for patients with similar typhoid/malaria symptoms in Germany and Togo [14].

But the reserved attitude towards autopsies is not uncommon, e.g. during the situation at the beginning of the discovery of the human immunodeficiency virus (HIV) [15].

The COVID-19 infected bodies showed very different disease courses with corresponding morphological and virologic findings. Details will be presented in further publications. The viral infection of the respiratory tract, the lungs and the associated acute respiratory distress syndrome have so far proved to be of central importance with respect to to fatal courses; however, the typical full picture of a single viral pneumonia is rarely developed. In addition, there are modifications due to superinfections or nosocomial infections, intensive therapy and long-term ventilation, occasional aspiration, various pre-existing conditions of the respiratory system and concomitant multimorbidity. In particular, an unusually high number of cases of ubiquitous deep leg vein thrombosis, recurrent pulmonary embolism and pulmonary infarction were found.

In the case presented, viral pneumonia with histologically determined diffuse alveolar damage, hyaline membranes and a hemorrhagic syndrome dominated. With respect to virologic findings, a highly positive throat swab was impressive. Similarly, viral RNA in the throat mucosa and lungs were found.

The clinical course of the man was typical with an incubation period of 3–5 days [16]. An infection right at the beginning of the journey or shortly after is assumed. There is no medical documentation available on the clinical findings in Egypt. The terminal course of the disease was unusually rapid [17]. The intensive treatment obviously consisted only of oxygen administration. Ultimately, decompensation of the cardiorespiratory system as a result of the lung affection occurred very rapidly within 1‑2 days. The considerable previous diseases, such as hypertension, cardiac hypertrophy and marked cor adiposum, identified by the autopsy may have contributed to the fatal outcome. As far as is known, these had not been recorded during previous medical examinations in Germany.

There are relatively few pathomorphological oriented studies on COVID-19 positive deceased [18–20]. The viral pneumonia is considered pathognomonic. The manifestation in other organs requires further studies. These are currently being conducted worldwide [21, 22].

## Conclusion

The worldwide SARS-CoV‑2 pandemic and the increasing number of COVID-19 related deaths show the emerging evidence for systematic autopsies on newly identified diseases as an important measure for the understanding of the pathomorphological findings and the clinical outcome of patients. To combine both research and also the approach of infectious diseases in forensic settings, the exact handling of the deceased during the external and internal post-mortem examination is crucial, using the safety levels of the TRBA and ABAS as a guidance.
